# Vertical exploration and dimensional modularity in mice

**DOI:** 10.1098/rsos.180069

**Published:** 2018-03-14

**Authors:** Yair Wexler, Yoav Benjamini, Ilan Golani

**Affiliations:** 1 Department of Statistics and Operations Research, Tel Aviv University, Tel Aviv, Israel; 2 Department of Zoology, Tel Aviv University, Tel Aviv, Israel

**Keywords:** vertical, rearing, climbing, exploration, mice, modularity

## Abstract

Exploration is a central component of animal behaviour studied extensively in rodents. Previous tests of free exploration limited vertical movement to rearing and jumping. Here, we attach a wire mesh to the arena wall, allowing vertical exploration. This provides an opportunity to study the morphogenesis of behaviour along the vertical dimension, and examine the context in which it is performed. In the current set-up, the mice first use the doorway as a point reference for establishing a borderline linear path along the circumference of the arena floor, and then use this path as a linear reference for performing horizontal forays towards the centre (incursions) and vertical forays on the wire mesh (ascents). Vertical movement starts with rearing on the wall, and commences with straight vertical ascents that increase in extent and complexity. The mice first reach the top of the wall, then mill about within circumscribed horizontal sections, and then progress horizontally for increasingly longer distances on the upper edge of the wire mesh. Examination of the sequence of borderline segments, incursions and ascents reveals dimensional modularity: an initial series (bout) of borderline segments precedes alternating bouts of incursions and bouts of ascents, thus exhibiting sustained attention to each dimension separately. The exhibited separate growth in extent and in complexity of movement and the sustained attention to each of the three dimensions disclose the mice's modular perception of this environment and validate all three as natural kinds.

## 1. Introduction

The open field test has been traditionally used to establish differences in locomotor and exploratory behaviour between rodent genetic, pharmacological, surgical and other experimental treatments [1–3]. In the common, forced, version of the test, the animal is introduced into a small experimental arena for a relatively short period of time, substantially reducing the potential of the test for studying exploratory behaviour [4,5]. To increase the test's potential and allow a study of the morphogenesis of exploratory behaviour from an ethological point of view, the size of the arena and the length of the experiment have been substantially increased, and the animal has been allowed to enter the arena deliberately from its home cage via a doorway [6,7]. Still, in both the free and the forced set-ups, exploratory behaviour in the vertical dimension, which is an integral part of these animals' behavioural repertoire, is severely curtailed, being limited to rearing and jumping [8–12]. At the other extreme, studies investigating the effect of drugs on vertical activity often prevent horizontal activity [13,14] and limit vertical movement by using relatively small boxes or cylinders [15–18]. A three-dimensional testing environment, better corresponding to the mouse's behavioural three-dimensional life-world [19–25] and therefore more appropriate for highlighting the mouse's cognitive and motor capabilities, is thus called for. In the present study, we expand the free open field set-up by attaching a vertical wire mesh to the arena wall, providing an opportunity to study the morphogenesis of vertical exploratory behaviour without limiting horizontal exploration. We accomplish this with hardly any additional costs (see Material and methods, figure 7).

The elementary particulate process of exploratory behaviour has been previously described as a ‘sequence of repeated motion’ [6,7]: an iterative process involving growth in extent and in complexity in reference to a specific origin. Sequences of repeated motion that were described so far include excursions from the home cage or its proximity [6,21] and incursions from the wall into the centre of the arena [6,26]. As elaborated previously [6], excursions first consist of movement along the wall (borderline movement), then differentiate to also include incursions, and then vertical movement in the form of jumping, covering each dimension before proceeding to the next one.

The addition of an affordable vertical dimension, embedded within a wide three-dimensional space, allows us to examine the robustness of the modular organization (borderline first, radial next and vertical last), and accomplish three main aims: (i) support and establish ascents on the wall as a new type of sequence of repeated motion by demonstrating their lawful growth in extent and complexity in reference to the ground, (ii) study the sequencing of ascents in the context of borderline movement and incursions and (iii) decipher the complexity of behaviour at advanced stages of the build-up of exploration, which thus far seemed to be performed in a haphazard way.

## 2. Results

### 2.1. Excursions grow in extent and in dimensional complexity across the session

**Figure 1.**
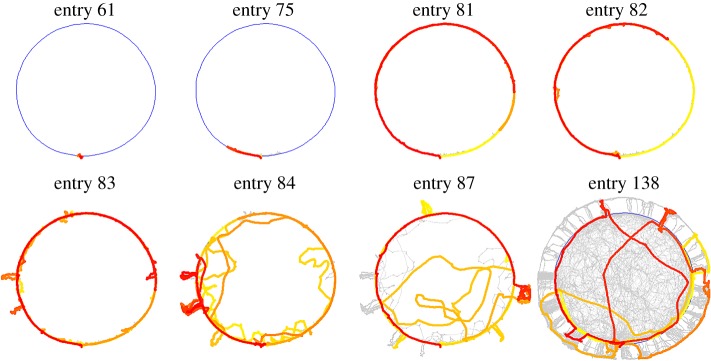
Eight selected exploratory entries from the home cage to the arena illustrate the growth along the three dimensions—borderline movement, radial movement (incursions) and vertical movement (ascents), across the session. The first entries consist of peeps, followed by small excursions that gradually grow in amplitude, covering the whole circumference. Then, the mouse performs incursions (centripetal forays from the circumference) and ascents (represented as centrifugal forays from the circumference). The doorway is located at the bottom of the circular arena. The directionality of the movement within an entry is marked by a transition from yellow (entry starts) to red (entry ends). Blue represents the arena circumference, and grey the path's history.

Figure 1 illustrates the gradual growth in the extent and complexity of the exploratory path across entries along the three dimensions of movement—borderline, radial (incursions) and vertical (ascents). An ‘entry’ starts when the animal enters the arena and ends with its departure, consisting of one or more excursions. Since borderline movement and incursions were extensively studied previously [6,7], we focus on ascents. In what follows, we first establish ascents as a type of sequence of repeated motion performed in reference to the ground, then we validate the role played by the ground as a whole as a reference location for ascents (rather than referring to particular locations on it), and lastly demonstrate the context in which ascents are exhibited in relation to the other dimensions—borderline movement and incursions.

### 2.2. Exploring the vertical dimension

We define an ascent as a segment of motion performed on the wire mesh, starting and ending on the ground. We characterize the growth in the extent and complexity of ascents in terms of five variables—the growth in ascents' (i) height (vertical amplitude), (ii) width (horizontal amplitude), (iii) complexity (estimated in terms of the number of direction changes), (iv) top vertical speed and (v) top horizontal speed. For each of these five variables, quantifying the growth was based on estimating a LOESS-smoothed 90% percentile function [7] (hereafter ‘percentile-LOESS function’) of the respective measure per ascent (see Material and methods). Peak growth was defined as the first ascent to reach a threshold of 80% of the maximum value (i.e. ‘time to reach 80%’). To establish a temporal order, all pairwise comparisons were performed (e.g. growth in height versus growth in width), in terms of the time to reach 80%. Significance was assessed using the Wilcoxon signed-rank tests. False discovery rate (FDR) at level 0.05 was controlled by the Benjamini–Hochberg (BH) procedure [27]. Therefore, unless specified otherwise, all *p*-values presented hereafter are adjusted accordingly, with values below 0.05 considered significant. All reported 95% confidence intervals for the differences in locations between pairs of variables are also FDR-adjusted [28].

Electronic supplementary material, Videos S1 and S2 illustrate the build-up across ascents in a single mouse, selected for performing the widest ascent.

### 2.3. Growth in ascents' height, width and complexity

**Figure 2.**
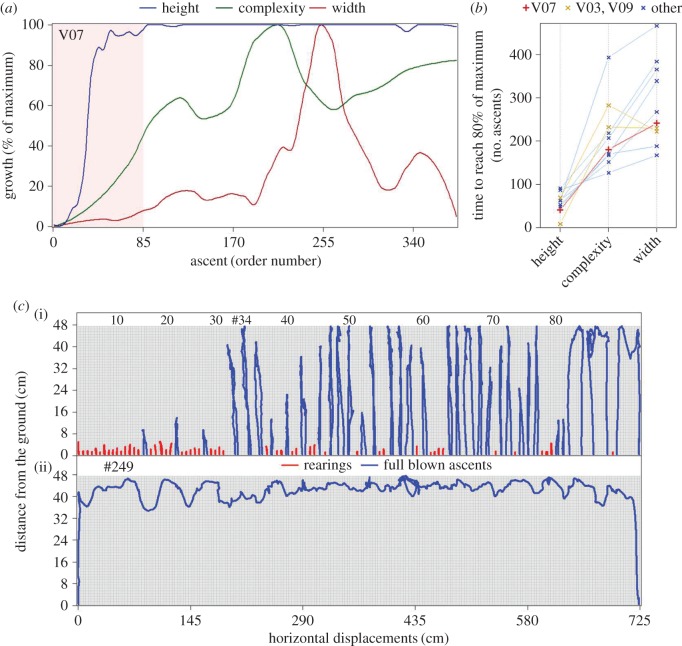
(*a*) Peak growth in height (dark blue) is followed by growth in complexity (green) and then by growth in width (dark red), as demonstrated by the 90th percentile-LOESS functions of one selected mouse (V07, selected for performing the widest ascent of all mice). The pink background marks the first 85 ascents, illustrated at *c*(i). (*b*) All mice reach peak growth in height (80% of maximum) before peak growth in complexity (*P*_adjusted_  =  0.0049; 95% CI of 146.5–303.0 ascents). All mice but two (V03 and V09, marked in gold) reach peak growth in complexity before peak growth in width (*P*_adjusted_ = 0.027; 95% CI of 6.5–143.5 ascents). Red marks mouse V07, whose session is used as an example in *a* and *c*, and blue marks all other mice. (*c*)(i) the paths traced on the wire mesh by mouse V07 (*a*, red line in panel *b*) during the first 85 ascents, presented side by side for simplicity, illustrate the growth in height, as well as the initial growth in width and complexity. Red marks rearing episodes, and blue marks ascents in which the mouse left the ground with all four limbs. Ascent #34 is the first to reach the top of the wire mesh. (ii) The path traced on the wire mesh by mouse V07 during the widest ascent (ascent #249, 720 cm wide).

As demonstrated in figure 2 and electronic supplementary material, Videos S1 and S2, ascents are preceded by rearing episodes on the arena wall (figure 2*c*, red segments). After repeated rearing, higher ascents, in which the mouse leaves the ground with all four limbs, are added to the repertoire (figure 2*c*, blue segments). These ascents are strictly vertical; the path downwards roughly traces the path performed on the way upwards. Eventually, the ascents reach the top of the wire mesh (figure 2*c*, ascent #34). The number of rearing episodes performed up to reaching the top of the mesh varies across mice (median of 36 ascents and interquartile range (IQR) of 30 ascents). Most important, as shown in the electronic supplementary material, Video S1, the rearing episodes and subsequent higher ascents are distributed across the whole perimeter of the arena.

With the growth in height exhausted, ascents start to grow in width—the mouse climbs vertically to the top of the wire mesh, then proceeds horizontally along the upper edge of the mesh, and then descends along a path parallel to the upward path. The tight attachment of the wire mesh to the wall does not allow the mice to walk on top of it, and therefore, horizontal movement (parallel to the ground) is performed as their ventral aspect faces the wall and the longitudinal axis of their trunk is roughly parallel to the ground.

The extent of the horizontal component of ascents gradually increases across the session. The width of the widest ascent across the 8 h session varies greatly between mice, with a median of 210 cm and an IQR of 350 cm. The widest ascent across mice was 720 cm wide (figure 2*c*, ascent #249), which is 92% of the arena circumference (785 cm).

As noted, horizontal movement is performed almost exclusively on the top portion of the mesh. The top quarter of the mesh accounts for a median of 71% of all horizontal displacement. This proportion is strongly correlated with the width of the widest ascent performed by the mouse (Spearman's *ρ* of 96%), implying that smaller proportions (minimum of 52%) were observed not because some of the mice favoured horizontal movement at the bottom of the wire mesh, but rather because they failed to perform wide ascents at the top.

Extending the horizontal component of ascents is initially accompanied by a gradually increasing freedom of movement, expressed by a growing number of direction changes between vertical and horizontal movement, 180° turns, 360° rotations, and forays from the top of the wall and back without touching the ground (descents). Changing direction is performed at low speeds, and high-speed motion segments of ascents are limited to strictly vertical or strictly horizontal movement. Diagonal movement up or down is rare. The growth in ascents' complexity accompanies the growth in width only in narrow to medium-width ascents (rightmost ascents in figure 2*c*(i)). Wide ascents, usually performed in the latter stages of the 8 h session, are characterized by long segments of a strictly horizontal movement, implying that the mouse's focus shifts from examining points of interest to covering as much of the top of the wall as possible.

As described and as demonstrated in figure 2*a*,*b*, growth in height reaches its peak before growth in complexity (*P*_adjusted_ = 0.0049; 95% CI of 146.5–303.0 ascents), and growth in complexity before growth in width (*P*_adjusted_ = 0.027; 95% CI of 6.5–143.5 ascents).

### 2.4. Notable outliers

In two out of 10 mouse-sessions, V03 and V09 (figure 2*b*, gold), the mice reached the peak in width before the peak in complexity. Both mice are further discussed below.

### 2.5. Growth in ascents' vertical and horizontal speeds

**Figure 3.**
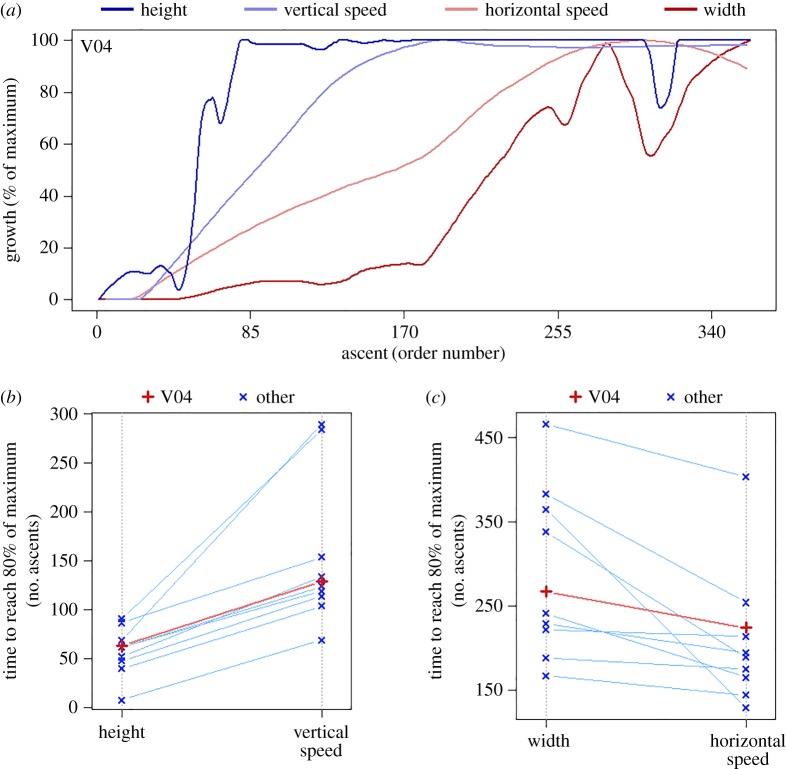
(*a*) In vertical movement on the wire mesh peak growth in amplitude (height, dark blue) precedes growth in speed (light blue), and in horizontal movement on the wire mesh peak growth in amplitude (width, dark red) follows growth in speed (pink), as demonstrated by the 90th percentile-LOESS functions of one selected mouse (V04, selected for faithfully representing the effect). (*b*) All mice reach peak growth in height (80% of maximum) before peak growth in vertical speed (*P*_adjusted_ = 0.0098; 95% CI of 61.5–143.0 ascents). (*c*) All mice reach peak growth in horizontal speed before peak growth in width (*P*_adjusted_ = 0.0049; 95% CI of 23.0–139.5 ascents). In (*b,c*), red marks mouse V04, whose session is used as an example in (*a*), and blue marks all other mice. No significant difference in time to reach the peak was observed between the vertical and horizontal speeds (*P*_adjusted_ = 0.14; 95% CI of −12.5 to 145.0 fewer ascents in vertical speed than horizontal speed).

As presented in figure 3, we compared the growth in vertical speed with the growth in height, and the growth in horizontal speed with the growth in width. As illustrated in figure 3*a* and demonstrated in figure 3*b*, in all mice growth in vertical speed lags behind the growth in height (*P*_adjusted_ = 0.0098; 95% CI of 61.5–143.0 ascents). Following the first arrival at the top of the wire mesh, subsequent arrivals are gradually faster. This suggests that as the session progresses, the wire mesh becomes a passageway between the floor and the top, rather than the object of exploration. Upward and downward vertical speeds are strictly coupled (i.e. both upward and downward top speeds increase and decrease together across ascents). However, in 95% of ascents top upward speed was higher than top downward speed (26% higher on average), suggesting that it is more difficult for the mice to go down than up. The temporal relation between growth in amplitude and growth in speed in vertical movement is reversed in horizontal movement (figure 3*a*,*c*)—in all mice horizontal speed reaches its peak before width (*P*_adjusted_ = 0.0049; 95% CI of 23.0–139.5 ascents). This reversal of order is presumably due to the fact that while vertical amplitude is bounded by the top of the wall, stopping the growth of the path before top vertical speed could be attained, horizontal amplitude is unbounded, and could potentially keep on growing even after the mouse reached the peak of its abilities in terms of horizontal speed.

Comparing the top vertical and horizontal speeds reveals that horizontal movement on the wall is considerably more difficult to perform at high speeds than vertical climbing. The mice reached top vertical speeds between 32 and 64 cm s^−1^, but the top horizontal speed reached was on average only 29.7% (s.e.m. of 1.9%) of the top vertical speed. No significant difference in time to reach the peak was observed between the vertical and horizontal speeds (*P*_adjusted_ = 0.14; 95% CI of −12.5 to 145.0 ascents in vertical speed than horizontal speed).

### 2.6. The lower circumference as a linear reference for ascents

As described, borderline movement on the floor (along the ‘lower circumference’) gradually increased in extent across excursions in reference to the doorway (point reference). Exploring the upper edge of the mesh (upper circumference) is accomplished by gradually increasing the extent of the horizontal component of the movement during ascents. However, in this case, the reference for the growth in extent is the entire lower circumference (linear reference), rather than a single point.

**Figure 4.**
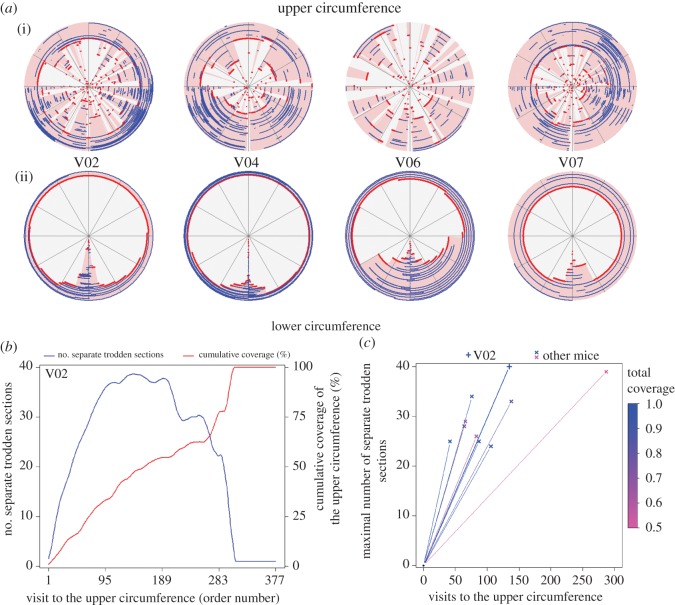
Exploring the upper circumference during ascents consists of opening numerous separate sections and then connecting them, whereas exploring the lower circumference during borderline movement consists of extending a single section to the left and to the right of the doorway. The pattern of exploration in the upper circumference indicates that the entire lower circumference is used as a linear reference for ascents. (*a*) Four selected mice sessions illustrate the difference in exploration patterns between the upper and lower circumference. For each ascent (i) or borderline excursion (ii), presented in the order of their performance from centre to periphery, red marks novel sections, blue marks repeated sections, and pink areas mark all previously covered sections. (*b*) In blue—the number of separate trodden sections per visit to the upper circumference (LOESS smoothed) of mouse V02. The function increases when a new section is opened and decreases when two or more trodden sections are connected. It is flat when a trodden section is either repeated or extended without being connected to another section. In red—the cumulative coverage of the upper circumference per visit (LOESS smoothed). (*c*) The mice open many separate trodden sections. The maximal number of separate sections across mice ranges between 24 and 40, and the average growth rate to maximum (the slope of each line) ranges between 0.14 and 0.60 separate sections per visit to the upper circumference. The colours of the lines indicate the total coverage of the upper circumference reached during each session.

Figure 4 and electronic supplementary material, Videos S3 and S4 illustrate the differences in the pattern of exploration of the upper circumference (figure 4*a*(i), ascents) and lower circumference (figure 4*a*(ii), borderline movement).

In borderline movement (figure 4*a*(ii)), the covered terrain consists of a single continuous section for each episode across the whole sequence of episodes. This section extends across the session to the right and left of the doorway, eventually covering the whole lower circumference. This pattern of growth in coverage characterizes growth in reference to a point location. It applies to all mice, and is illustrated in the four selected animals in figure 4*a* and in electronic supplementary material, Video S3. In ascents (figure 4*a*(i)), successive episodes generate new additional sections of trodden paths, all around the upper circumference. With time, trodden sections are extended and connected to other trodden sections. As illustrated in figure 4*a* and in electronic supplementary material, Video S4, while all mice commence with narrow sections that spread sporadically all around the circumference (central red dots), mouse V02 develops a preference for the bottom right quarter (concentration of concentric peripheral blue lines), V04 develops a preference for the bottom left quarter, V06 shows no clear preference and V07 develops a preference for the top right quarter. The individual preference for specific sections highlights the self-assembly nature of emerging habits. For mouse V02, selected for covering the whole upper circumference, the dynamics of opening, extending and connecting trodden sections on the upper circumference are presented in figure 4*b*. This mouse opened 40 separate sections in the first 135 visits to the upper circumference (average growth rate to maximum of 0.30 separate sections per visit), and then proceeded to connect them until it eventually covered the whole circumference. The maximum number of separate trodden sections varied across mice, ranging between 24 and 40, with the average growth rate to the maximum ranging between 0.14 and 0.60 separate sections per visit to the upper circumference (figure 4*c*). The growth rate after the first 10 visits to the upper circumference, however, was much higher, ranging between 0.5 and 0.8. The steep initial growth rate and the large maximal number of separate trodden sections are inconsistent with a strategy involving a limited number of point references, implying that the mice use the entire lower circumference as reference for ascents.

### 2.7. Notable outliers

Previously, two mice, V03 and V09, were mentioned as outliers for reaching peak growth in ascents' width before peak growth in complexity (figure 2*b*, gold lines). For mouse V03, the reversed order could be explained by the relative lack of ability to extend the width of ascents. Even though it performed the largest number of ascents, the widest ascent was 66 cm wide—the narrowest among all mice (average of 3 m). Furthermore, it only opened and repeated trodden sections, hardly ever connecting them. Consequently, mouse V03 exhibited the lowest growth rate to the maximum (0.14, rightmost point in figure 4*c*) and covered the smallest portion of the upper circumference across mice (52%). Mouse V09 was unusual in its behaviour both on the ground and on the wire mesh, characterized in an exceptionally high growth rate in all three dimensions—borderline movements, incursions and ascents. This mouse also exhibited the highest growth rate to the maximum number of separate sections (0.60, leftmost point in figure 4*c*), and was the fastest to cover the whole upper circumference. It did demonstrate growth in complexity which accompanied the initial growth in width, but the peak number of direction changes was achieved after the widest ascent was performed.

### 2.8. The build-up of movement in three-dimensional space

Having used growth and differentiation to support the notion of ascents as a sequence of repeated motion, we now set to determine the context in which ascents are exhibited in relation to the other dimensions. Fonio *et al*. [6] claim that the developmental sequence of free exploration in an arena without a vertical wire mesh attached to the wall involves an ordered emergence of the three dimensions of movement (borderline first, radial next and vertical last) and an exhaustion of each dimension before the emergence of the next one. In that set-up, the invasion of the vertical dimension takes the form of jumping. They also point out that ‘there is always a build-up in amplitude within each dimension’. The addition of the wire mesh in the present study provides an opportunity to examine the robustness of the rules exhibited in the wireless arena.

**Figure 5.**
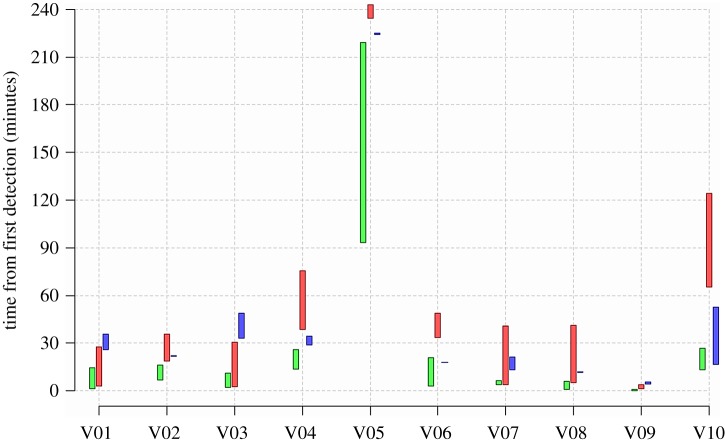
While borderline movement (green bars) emerges before incursions (red) and ascents (blue), the order of emergence of incursions and ascents varies across mice. Also, most of the mice exhibit emergence of a new dimension prior to the exhaustion of the previous one. The bottom of each bar represents the time (in minutes) to start the first episode in the respective dimension, and the top of the bar represents the time to reach an advanced developmental landmark.

Figure 5 presents for all mice the timing of emergence and exhaustion of each dimension—borderline movement (green), incursions (red) and ascents (blue). Emergence, whose timing is represented by the bottom of each vertical bar, is measured by the time, in minutes, from the first detection of the mouse in the arena to the start of the first full-blown sequence—of moving away from the doorway in borderline movement, from the wall in incursions and from the ground in ascents; peeps and rearings are not scored. Exhaustion, represented by the top of each bar, is measured in terms of the time to reach full coverage of the lower circumference in borderline movement, the centre of the arena in incursions and the top of the wire mesh in ascents.

With the exception of one animal, the borderline movement is the first to emerge and be exhausted. However, the temporal order of emergence of incursions and ascents varies across mice. In six of the mice incursions emerge before ascents, and in the other four, the order is reversed. This presumably reflects the relatively high incentive value [29] for climbing on the wire mesh compared to that for jumping on the smooth wall, which was used in Fonio *et al.* [6].

As described in previous subsections of the Results section, reaching the top of the wire mesh is only a landmark in the build-up of ascents, marking the end of the growth in height, rather than a true indication of exhaustion. The same could be argued for incursions and reaching the centre of the arena. Therefore, using these two landmarks in this context may provide a rather shaky basis for comparing the timing of performance of incursions and ascents. It does reveal, however, in most mice, an overlap between dimensions which should not have existed had the emergence of a new dimension required the exhaustion of the previous one.

Therefore, as demonstrated, neither the order of the developmental sequence nor the exhaustion of each dimension before proceeding to the next one hold in the set-up including the vertical wire mesh.

The claim that there is always a build-up in extent within each dimension does, however, apply to the current experimental set-up, having been demonstrated in the present study for ascents (figures 2 and 3), and for borderline movement and incursions (electronic supplementary material, figure S1).

The observation that the three dimensions overlap in the moment-to-moment developmental sequence does not necessarily imply that the transition across dimensions is haphazard: in the rest of this subsection, we examine the nature of the transition across dimensions, establishing that incursions, and separately, ascents tend to be performed in bouts.

**Figure 6.**
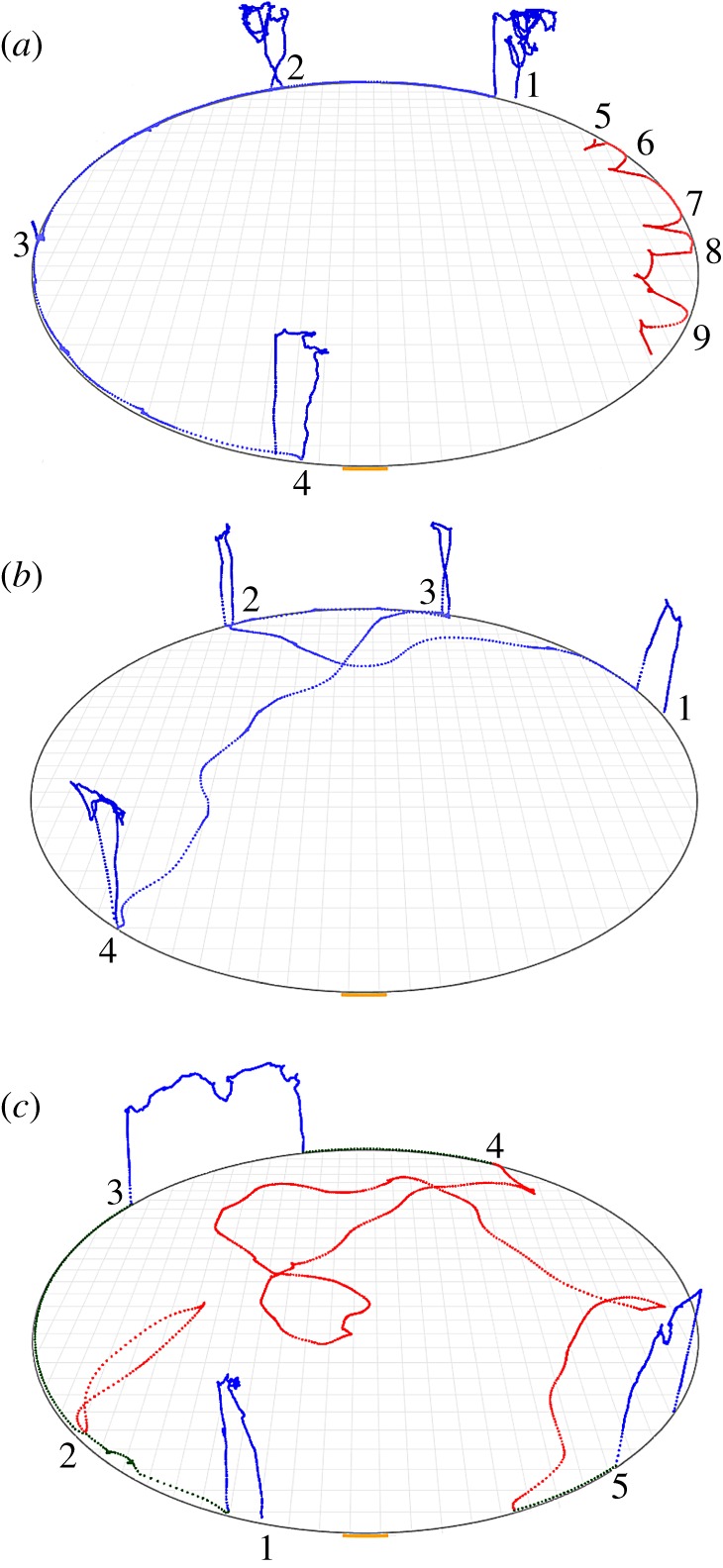
Schematic of bouts of ascents (in blue) and incursions (in red). (*a*) Direct transitions between ascents or incursions via borderline movement. In blue—a bout of four successive ascents, labelled 1–4 in the order of performance, separated by borderline movement (in light blue). In red—a bout of five successive incursions, labelled 5–9, separated by borderline movement (in light red). (*b*) Direct transitions between ascents via direct incursions and borderline movement. In blue—a bout of four successive ascents, labelled 1–4. Ascents 1 and 2 are separated by a single direct incursion (in light blue), and so are 3 and 4. (*c*) Alternating single ascents (in blue) and single incursions (in red), labelled 1–5. Borderline movement is not associated with any bout and is coloured in green.

As detailed in Fonio *et al.* [6], the session begins in all mice with several entries consisting exclusively of borderline movement. As the session progresses, incursions and ascents emerge, and all three types of movement intertwine. We define two successive ascents within the same entry as belonging to the same bout of ascents if they are connected to each other by a ‘direct’ transition on the floor. A similar definition is applied to two successive incursions. A bout of movements may thus consist of at least two successive movements belonging to the same dimension. The difference between direct and indirect transitions is illustrated in figure 6, using three schematic types of series of ascents and incursions.

In thigmotactic mice [6,30], the shortest path between two locations on the borderline is borderline movement. Therefore, a borderline transition between two successive ascents is treated as direct, and the two ascents are then considered as belonging to the same bout (figure 6*a*, blue section). Two successive incursions connected by borderline movement are similarly treated as belonging to the same bout (figure 6*a*, red section). If the transition between two ascents consists of two or more incursions, it is considered an indirect transition. These ascents then belong to different bouts.

Examination of the tortuosity of single incursions reveals that the population of these path segments consists of a mixture of two distinct types: those involving a relatively direct transition from start to end and those involving an indirect, tortuous transition (see Material and methods). Successive ascents connected by a single direct incursion (with or without borderline movement) belong to the same bout (figure 6*b*), whereas ascents connected by a single indirect incursion do not belong to the same bout. Figure 6*c* illustrates a series of alternating single ascents (in dark blue) and single indirect incursions (in dark red).

The size of a bout is determined by the number of ascents, or, respectively, incursions, included in it (figure 6*a*,*b*). The alternation of single ascents and single incursions are obviously not regarded as a bout. The maximal bout size in ascents ranged across mice from 6 to 12 ascents, with an average of 9. The maximal bout size in incursions ranged across mice from 6 to 16 incursions, with an average of 11.2.

To test the hypothesis that mice prefer to perform bouts against the null hypothesis of random alternation between ascents and incursions, we performed one-sided permutation tests on all the mouse-sessions (see Material and methods). The 10 *p*-values were adjusted using the BH procedure [27] to control FDR at level 0.05. With nine out of 10 adjusted *p*-values under 0.05 (maximum of 0.005), we can reject the global null hypothesis and conclude that BALB/c mice tend to organize their performance of ascents and, respectively, incursions, in bouts. These mice performed between 20 and 88 single incursions and ascents fewer than the averages of their respective permutations distributions. Mouse V09, a recurring outlier mentioned previously regarding the comparison of growth in complexity and width, performed 14 single incursions and ascents below distribution average, and for which the observed adjusted *p*-value was 0.094.

Electronic supplementary material, Videos S5 and S6 illustrate that bouts are indeed the strategy preferred by the mice to organize the occupancy of the three-dimensional space. Electronic supplementary material, Video S5 is set in a relatively early stage of the session of mouse V01—entries 29 and 30, which are the first to include full-blown ascents. Electronic supplementary material, Video S6 is set over an hour and a half later—entries 62 and 63. In both videos, bouts of ascents are coloured in blue, bouts of incursions in red and borderline movement not associated with any bout in green. In electronic supplementary material, Video S5, there is a clear separation between ascents and incursions, with all but one direct transition between ascents consisting entirely of borderline movement (figure 6*a*). In electronic supplementary material, Video S6, set at a stage where the mouse has already exhausted the horizontal plane of the arena, the separation between ascents and incursions is diminished, and direct transitions between ascents featuring single direct incursions (figure 6*b*) are exhibited regularly.

Examining the emergence of bouts of incursions and ascents reveals that borderline movement is also initially performed in a bout. As mentioned, all mice begin the session with a series of borderline-exclusive excursions. In nine out of 10 of the mice, this series is interrupted by one excursion containing a single incursion or a single ascent, before another series of borderline-exclusive excursions is performed. Joined together, these two series of excursions may be viewed as a single bout of borderline movement, which ends with the emergence of bouts of size 2 and larger, of incursions and/or ascents. Furthermore, eight out of the 10 mice cover the whole lower circumference in the initial bout of borderline movement, which varies greatly in size, ranging from 4 to 185 excursions. With the appearance of bouts of incursions and ascents, hardly any further bout of borderline movement is performed.

The performance of bouts as an integral part of the build-up in excursions supports the view that exploratory behaviour is modular, consisting, in this set-up, of three distinct components (dimensions), all of which grow and differentiate independently of each other.

## 3. Discussion

This study accomplishes several aims: (i) By demonstrating that ascents grow and differentiate in a regular, lawful way in reference to the ground during the occupancy of a novel environment we validate ascents as a ‘sequence of repeated motion’ [6,31], a natural particulate process of exploration, what Quine defined as a natural kind [32,33]. (ii) By demonstrating that in a newly designed environment, dimensional modularity is a robust phenomenon, exhibiting resistance to fragmentation, we support the view that vertical behaviour has a ‘physiological’ reality, i.e. the physical vertical dimension is not mapped, or enacted, haphazardly, but rather through sustained durable dimension-specific attention, endowing it with a unity in the mouse's operational world. (iii) By demonstrating the performance of bouts deep into the session, at a stage previously considered by us to be unpredictable or free, we deciphered the complexity of behaviour at an unprecedented late stage in our analysis.

### 3.1. Growth and differentiation

In this study's view, behaviour is a continuous well-connected morphogenetic process starting with the very first peeping into the arena and culminating, across a several-hour session in a mixture of bouts of types of repeated motion performed iteratively along three ‘physiological’ dimensions. The third, vertical, dimension grows and differentiates through an iterative process which follows specific rules, starting with the first rearing along the wall, and culminating in full-blown ascents including runs on the upper circumference of the wire mesh (electronic supplementary material, Video S1). This finding is added to previous demonstrations of growth and differentiation of exploratory behaviour, in both egocentric and allocentric space. In egocentric space, growth and differentiation has been shown in the build-up in the movement of the parts of the trunk (the so-called ‘mobility gradient’) [21,22,29,34]. In allocentric space, growth and differentiation have been demonstrated previously in the build-up of the exploratory path traced in the horizontal domain [6,7,35]. The morphogenetic process thus applies not only to anatomy, but also to behaviour, where both growth and differentiation offer an opportunity to observe the transition from simple to complex, and the gradual build-up provides a view on the organization of behaviour.

### 3.2. Exploring the vertical dimension

During exploration of the vertical dimension, the mouse first reaches the top of the wire mesh (upper circumference), then mills about at the top within circumscribed horizontal sections, and then progresses relatively monotonically for increasingly longer distances on the upper circumference. Once reached, the upper circumference becomes the focus of exploration, and the wire mesh itself then becomes a passageway between the floor and the top. The movement on the wire mesh is either strictly vertical or strictly horizontal (with the mouse's ventral aspect facing the wall and the longitudinal axis of the trunk roughly parallel to the ground). Diagonally oriented movement is exhibited rarely on the wire mesh, and it could not be attributed to the orientation of the wires (see Material and methods).

### 3.3. Sprouting of movement along new dimensions

The milling about at the upper circumference consists of incipient movements in both horizontal directions, which precede the growth in width. Similar incipient movements in the form of rearing episodes precede the emergence of the stage of climbing on the wall. Similar incipient movements also precede the emergence of excursions and the emergence of incursions [6]. In other words, much like the budding of an organ in organogenesis, a period in which the mouse performs ‘buds’ of movement announces the emergence of full-blown movement (and attention) along a new dimension.

Reminiscent of the ‘vicarious trial and error’ reported to be performed by rats at the choice point of a maze, as they would pause and look back and forth as though confused about which way to go [36–38], imagining potential future options, or looking into memories [38], are beyond our ethological scope, which focuses on structural growth and differentiation.

### 3.4. Linear reference for movement

In this study, we show that the mice first use the doorway as a point reference for establishing familiarity with the lower circumference; once established, the lower circumference is used as a line reference for the performance of incursions and ascents. A similar situation of hierarchically nested references has been reported in the exploration of a room by an infant rat during repeated daily exposures [39]: the infant first establishes a home base in a specific corner, using subsequent corners as secondary home bases to which it returns frequently while also returning from time to time to the first main home base. This is perhaps why the mouse exploring the upper circumference first relates to the lower circumference and not to the doorway.

### 3.5. Modularity

While bouts are commonly defined in the study of behaviour on the basis of how short are the time intervals connecting their constituents [40], we define a bout on the basis of how spatially direct (un-tortuous) is the transition between them. Having shown that the bouts are not assembled fortuitously, we consider them to reflect modularity: each bout discloses sustained attention to its respective dimension, and movement along each dimension grows and differentiates separately.

While in the novel experimental set-up, absolute, ordered (borderline first, incursions second, ascents last) complete (exhaustion of a dimension before shifting to the next dimension) modularity is disrupted, partial modularity is preserved: the dimensionality of the mouse's attention has not been fragmented into single ascents and single incursions by the present experiment, as single occurrences of these motion types are relatively rare. Is there a conceivable environment where attention would drift haphazardly across dimensions, or is the modularity of attention an endogenous constraint on exploratory behaviour? Could the degree of modularity be regulated by controlling the attractiveness of the substrates of the different dimensions?

### 3.6. An ethologically relevant setting

A set-up including simulation of ‘moonlight’, provision of familiar shelter with ad libitum access to food and water, as well as adequate resources of time and space, all provide optimal conditions for a spread-out build-up of deliberate exploration. The results provide a baseline which can now be used to examine any effect, such as rearing conditions, different lighting, food deprivation, genetic differences, drugs and lesions. The examination of different lighting is, however, limited, given the requirement for deliberate exploration (out of a home cage), without which the phenomena described in this study could not be exposed. When the arena is lit, the mice typically do not come out of the dark home-cage for days (on the other hand, full-blown exploratory behaviour including the establishment of a home base has been demonstrated in rats under different lighting conditions, including complete darkness, in an arena with no home cage [41]).

### 3.7. A behavioural trap

During ascents involving release of contact with the ground, all mice without exception descended to the ground with their heads leading. While most of the mice pivot at the top of the first of these ascents and readily descend, some pivot repeatedly without descending, and then commence to progress either upwards or sideways, pivoting again etc. This sequence may be repeated many times with increasing amplitude until the mouse eventually extricates itself from the wall. It is as though these mice ‘get trapped’ on the wall. This phenomenon disappears in subsequent ascents, as the mouse resumes the gradual build-up along the vertical dimension. The phenomenon is reminiscent of the ‘behavioural traps’ reported in recovery from akinesia in lateral-hypothalamic rats [42], where it has been interpreted as reflecting a dimension-specific deficiency overcome by gradual build-up along that dimension. Horev *et al*. [43] report a similar deficiency characterizing descents in mice harbouring deletion of the chromosomal region corresponding to 16p11.2, associated with autism. It might prove useful, therefore, to examine mouse models of autism for deficiency in the build-up of the first descents, expressed in our set-up by exceptionally extended ascent durations (electronic supplementary material, figure S2).

## 4. Material and methods

### 4.1. Animals, experimental set-up and testing protocol and analysis

In this and in previous studies, we used mice of the BALB/c strain because they are known to exhibit neophobia, providing a slow and spread-out version of the behavioural build-up which has been the focus of the present study. Years of experience with breeding and raising both laboratory and wild mice have taught us that the effect of neophobia on the build-up of locomotor behaviour overrides the role played by the environment in which the mice were brought up. In other words, the build-up in behaviour on the wire mesh reflects neophobic constraints, rather than learning how to climb.

**Figure 7.**
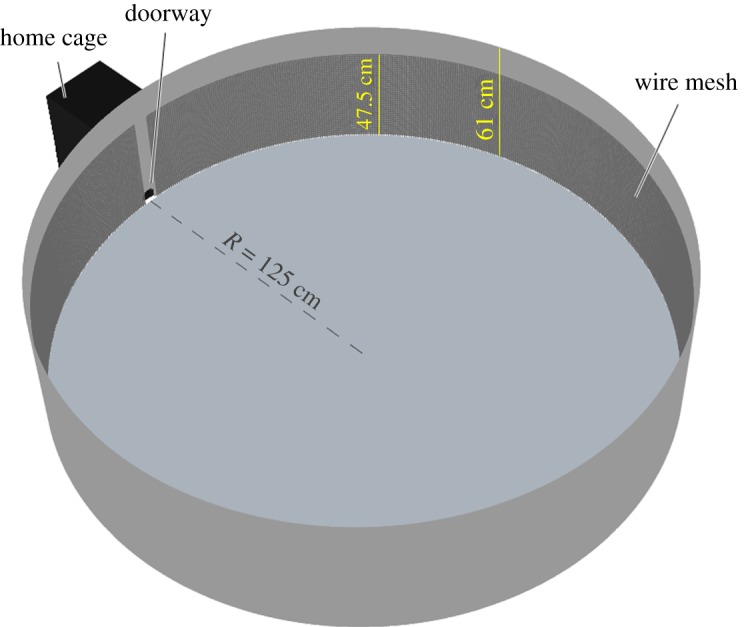
Illustration of the experimental set-up. A single video camera and two infrared lights are located directly above the centre of the arena. The vertical wire mesh is tightly attached to the circular wall of the arena. The tight attachment of the wire mesh to the wall does not allow the mice to walk on top of it, and therefore horizontal movement (parallel to the ground) is performed as their ventral aspect faces the wall and the longitudinal axis of their trunk is roughly parallel to the ground.

The BALB/c mice (*n* = 10 males, 11 weeks of age; Envigo laboratories) were kept in a 12 L : 12 D light cycle (light from 21.00 to 09.00 h). They were housed for two weeks in standard cages in groups of four and six animals, respectively, belonging to the same litters, at 22°C with water and food ad libitum. The enhanced DIEM assay [6] used for this experiment consists of a circular arena with a 2.5 m diameter and a home cage (figure 7). The walls of the arena are covered by a 47.5 cm high wire mesh, where the size of the holes is 1 cm by 1 cm. To examine the effect of wire mesh orientation on the direction of climbing, we performed preliminary observations in which we varied the orientation of the wires from parallel to the ground to diagonal, we found that in both conditions, the mice ascend and descend vertically. In other words, a diagonal orientation of the wires does not induce diagonally oriented ascents. We therefore used the vertical–horizontal orientation which is easier to construct and handle.

Each trial consisted of a 24 h long adaptation period, in which the mouse was closed in the home cage with food and water, and 8–24 h filming period which began with opening the door between the cage and the arena at 9 a.m. Both adaptation and filming periods took place in IR light only. The trials were filmed using a single overhead camera. Offline two-dimensional tracking of the films was done with Ethovision XT [44], data preparation was done with SEE, including smoothing of the tracking coordinates and estimating velocities with LOESS, and capturing intervals of zero velocity with repeated running medians [45–47]. Further analysis was done using R.

### 4.2. Transformation from two to three dimensions

Transformation from a two-dimensional to a three-dimensional coordinates system was based on estimating a border between the arena floor and the wall, using a modified version of the algorithm for estimating the boundary of the arena presented by Sakov *et al.* [47]. In a perfectly circular arena with known radius and centre, the distance of the animal from the wall is directly computed from the distance to the centre. In practice, an arena might have some deviations from a perfect circle (sometimes hardly noticeable to the eye). The effect of such deviations on distance from wall, if computed under the assumed perfect circle, might be devastating, for example, for capturing rearing episodes along the wall and tiny incursions towards the centre. Adopting high standards of data preparation for analysis to ensure replicability of results across laboratories (http://www.replicability.tau.ac.il/index.php), the algorithm, named ‘arena builder’ and designed for a two-dimensional set-up, exploits the fact that the mice spend a considerable amount of time along the arena border, marking clearly the borderline. This behaviour is used to correct at the software level the physical defects in the circularity of the arena [47]. In the three-dimensional set-up, arena builder cannot be used as is, as it would estimate the top edge of the wire mesh instead of the boundary between the floor and the wall. To separate between the floor and the wall, we used the fact that the mice run faster by an order of magnitude on the floor along the wall compared to the horizontal component of their movement on the wire mesh. In polar representation, both the horizontal component of movement on the wire mesh and movement on the floor along the wall are represented by tangential speed, and so high tangential speed above a customized threshold (see below) marks the boundary between the floor and the wall (electronic supplementary material, figure S3).

The modified arena builder divides the circular arena into *S* sectors, and calculates, for each sector *s* ∈ *S*, the distance *R_s_* of the boundary from an estimated centre of the arena. Let (*r_i_*, *θ_i_*) be the polar representation of the animal's smoothed Cartesian coordinates at time *i*, using the estimated arena centre as origin. Let $V_{\theta_{i}}=r_{i}(\text{d}\theta_{i}\text{/d}t)$ be the tangential speed at time *i*, which represents horizontal speed on the wall. An upper bound $V_{\theta \_\text{max}}$ is estimated for the tangential speed on the wall. The set {*R_s_* | *s* ∈ *S*} is then calculated using only the set of coordinates $\left\{(R_{i},\theta_{i})|\;\;V_{\theta_{i}}>V_{\theta \_\text{max}}\right\}$, where the animal has reached high tangential speed.

Having established the border between the arena floor and the wall, the mouse's coordinates at time *i* are transformed from polar (*r_i_*, *θ_i_*) to cylindrical $\left({\tilde{r}}_{i},{\tilde{\theta }}_{i},{\tilde{z}}_{i}\right)$. If at time *i* the animal was traced in sector *s*, then ${\tilde{r}}_{i}=\text{min}\{r_{i},R_{s}\}$, ${\tilde{\theta }}_{i}=\theta_{i}$ and ${\tilde{z}}_{i}=\text{max}\{0,h\cdot (1-(R_{s}\text{/}r_{i}))\}$, where *h* is the height of the overhead camera (2 m from the ground). Ascents were defined as episodes where $\tilde{z}\geq 1\;\text{cm}$.

### 4.3. Quantifying and statistical analysis of growth

The values of five kinematic variables were estimated per ascent—(i) maximal height, (ii) maximal width, (iii) complexity, (iv) top vertical speed and (v) top horizontal speed. Top vertical and top horizontal speeds per ascent were defined as the 95th percentile to avoid outliers. Complexity was estimated in terms of the number of direction changes (see below). To compare the growth between the variables, all measurements were scaled to a percentage interval (where 100% represents the maximum value per measure). The dynamics of growth were captured in each of the five kinematic variables by estimating an LOESS-smoothed 90th percentile function of the respective value per ascent. The algorithm first calculates moving percentiles (90%) using a fixed window width *w_p_*, then fits an LOESS curve to the moving percentile output, using a second fixed window width *w*_LOESS_. Both windows represent the number of ascents. This process is illustrated in electronic supplementary material, figure S4. For each kinematic variable, the two window sizes *w_p_* and *w*_LOESS_ were fixed for all 10 mice. However, different window sizes were used for each of the five variables. For example, for all mice, we used *w_p_* = 11 and *w*_LOESS_ = 30 for height and *w_p_* = 11 and *w*_LOESS_ = 80 for width. Window widths for the rest of the variables were *w_p_* = 50 and *w*_LOESS_ = 50 for top vertical speed, *w_p_* = 50 and *w*_LOESS_ = 50 for top horizontal speed and *w_p_* = 11 and *w*_LOESS_ = 70 for complexity. Window overlap for the moving percentile function was set to 90%. The starting values were padded with zeroes the length of two times *w_L_*.

Comparing the growth in the extent and complexity of ascents was based on the time (in number of ascents) to reach 80% of the maximum value. Comparing this endpoint across variables determines the order in which they grow to maximum. However, because each of the five variables is subject to different constraints a threshold of 100% is unsuitable. For example, the growth in ascents' height is cut short abruptly when the mouse reaches the top of the wall. By contrast, the growth in top vertical and in top horizontal speeds, which are not bounded by the shape of the arena, enter a stage of ‘saturation’ above 80% (illustrated in figure 2*a*). The threshold of 80% is also equivalent in distance from the top of the wall to approximately the length of two mice (9.5 cm). Therefore, we use this threshold as it reflects a maximal point where growth is full blown and relatively unaffected by uneven constraints.

Significance of differences in time to reach 80% across the five variables of growth was assessed using the Wilcoxon signed-rank tests. *p*-values were adjusted by the BH procedure [27] controlling FDR at level 0.05. All reported 95% confidence intervals for the differences in locations between pairs of variables are also FDR-adjusted [28].

### 4.4. Estimating direction changes as a measure of complexity

As mentioned in the Results section, the movement of the mice on the wire mesh is typically either strictly vertical or strictly horizontal. Therefore, estimating the number of direction changes in a single ascent is based on counting the number of transitions between vertical and horizontal movement on the wire mesh (90° turns), as well as the number of 180° turns in each axis. However, because a strict vertical movement would have some small horizontal component and vice versa, isolating turns required confining the mouse's movement to a single axis. The algorithm, applied to each ascent, involves two iterations, illustrated in electronic supplementary material, figure S5. The first iteration comprises (i) comparing the vertical speed and the horizontal speed at each time point—the higher of the two is kept, and the lower is set to zero, (ii) reconstructing the vertical and horizontal coordinates of the mouse on the wire mesh throughout the ascent by integration of the new ‘constrained’ vertical and horizontal speeds (electronic supplementary material, figure S5*b*), (iii) smoothing the reconstructed coordinates with repeated moving average (two repetitions with window width of 1.6 s, electronic supplementary material, figure S5*c*) and (iv) reconstructing the point vertical and horizontal speeds by differentiating the smoothed coordinates. The second iteration repeats steps (i) and (ii) of the first iteration (electronic supplementary material, figure S5*d*), and then 90° and 180° turns are easily counted.

### 4.5. Direct and indirect transitions between successive ascents

**Figure 8.**
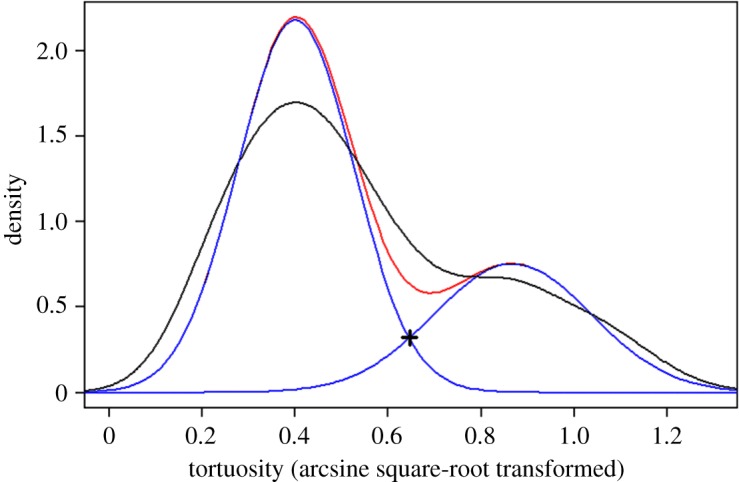
There are two distinct types of incursions—direct and indirect, as demonstrated by a histogram of the tortuosity (undergone arcsine square-root transformation) of the incursions performed by mouse V01. The blue lines represent the density of the two Gaussians fitted using the EM algorithm. The red line represents the density of the fitted mixture. The black ‘+’ represents the Gaussian intersection. Incursions whose transformed tortuosity is lower than the intersection are classified as direct, and incursions whose transformed tortuosity is greater than the intersection are classified as indirect.

As detailed in Results, several successive ascents or, respectively, incursions are grouped together in bouts if the transition between them is ‘direct’. As illustrated in figure 6*b*, two successive ascents are considered as belonging to the same bout if the transition between them contains no more than one direct incursion. Classification of incursions to direct and indirect is based on the complement of the so-called ‘straightness index’ [48,49] as a measure of the tortuosity of the path. If *D* is the distance between the starting point and the ending point of an incursion, and *L* is the length of the path travelled by the mouse in that incursion, then the straightness index is *D*/*L*, and the tortuosity is 1*−D*/*L*. As illustrated in figure 8, for all mice separately a Gaussian mixture model was fit to the tortuosity (undergone arcsine square-root transformation) using the expectation-maximization (EM) algorithm [50,51], which estimates the maximum-likelihood parameters (proportions, means and standard deviations) of a mixture with a given number of components (Gaussians). The number of components in the mixture model was determined by a likelihood ratio test, comparing the maximum likelihood of an *n*-components mixture to that of an (*n*−1)-components mixture. A detailed review of the process is given by Drai *et al*. [51]. For all mice but one, the number of components determined was two. For mouse V02, a mixture model with three components was used. The intersection between the density functions of the leftmost (direct, low tortuosity) and rightmost (indirect, high tortuosity) Gaussians was used as the tortuosity threshold. Therefore, two successive ascents, between which the transition contains a single incursion, were considered as parts of the same bout if the tortuosity of that incursion was lower than the threshold (after the reversed sine squared transformation).

### 4.6. Establishing the tendency of the mice to perform bouts

To test the hypothesis that mice prefer to perform bouts against the null hypothesis of random alternation between ascents and incursions, we performed one-sided permutation tests on all mouse-sessions. For each mouse, we permuted the order of performance of incursions and ascents in the entire session, divided the permuted sequence into entries and then grouped successive ascents, and, respectively, incursions, in bouts. The test statistic was the total number of single ascents and incursions in the session, and the *p*-value was the number of permutations (out of 100 000) to have the same number or fewer than the original sequence, assuming exchangeability under the null hypothesis. The 10 *p*-values were adjusted using the BH procedure [27] to control FDR at level 0.05.

## Supplementary Material

Figure S1. Separate growth in excursions, incursions and ascents

## Supplementary Material

Figure S2. Example for a behavioural trap in which the mouse “gets stuck” on the wall

## Supplementary Material

Figure S3. High tangential speed indicates movement on the floor

## Supplementary Material

Figure S4. Percentile-LOESS function estimates the growth in ascents' height

## Supplementary Material

Figure S5. Estimating the number of direction changes in an ascent

## Supplementary Material

Supplementary videos

## Data accessibility

Our data and code are deposited at Dryad: (https://doi.org/10.5061/dryad.t29p3) [52]. The R code contains the modified arena builder, transformation to three-dimensional coordinates and analysis.
