# Supplementary figures and images for: Vertical exploration and dimensional modularity in mice
Source: R Soc Open Sci. 2018 Mar 14;5(3):180069. doi: 10.1098/rsos.180069 (PMC5882751; doi:10.1098/rsos.180069)

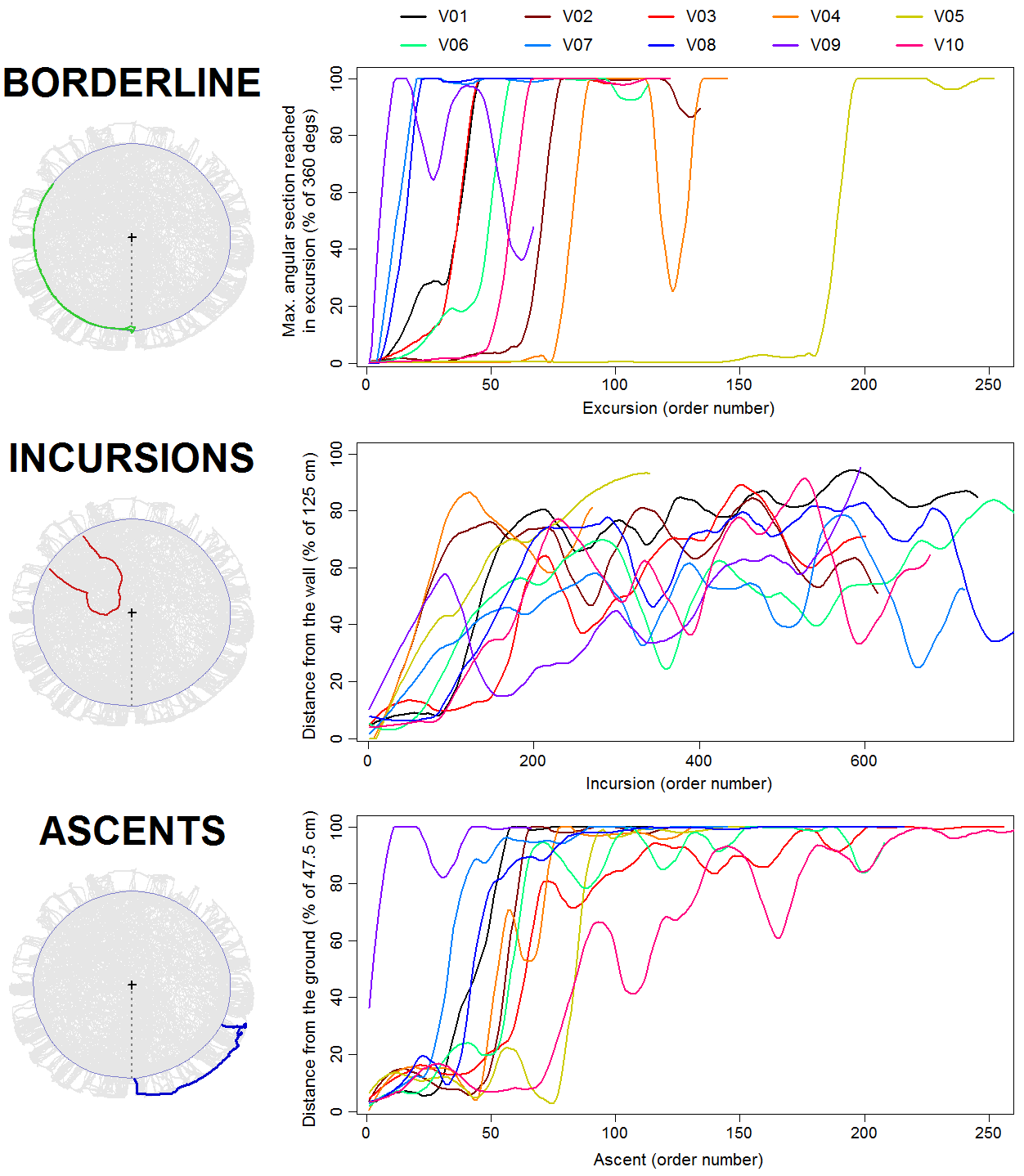

Supplement: Figure S1. Separate growth in excursions, incursions and ascents [file rsos180069supp1.tif]

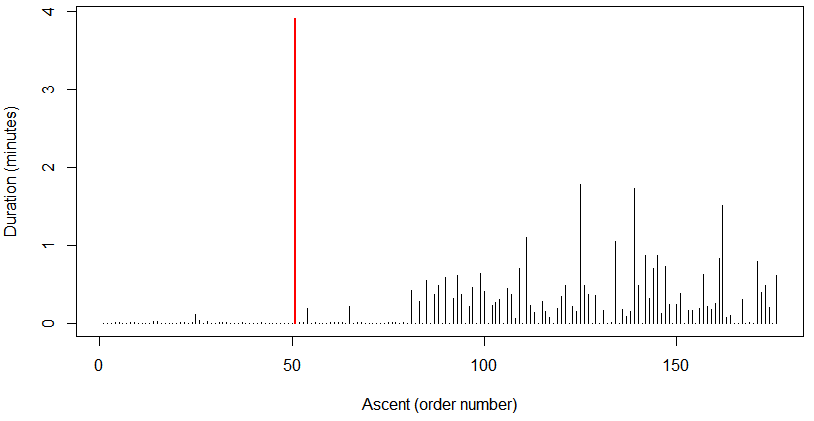

Supplement: Figure S2. Example for a behavioural trap in which the mouse “gets stuck” on the wall [file rsos180069supp2.tif]

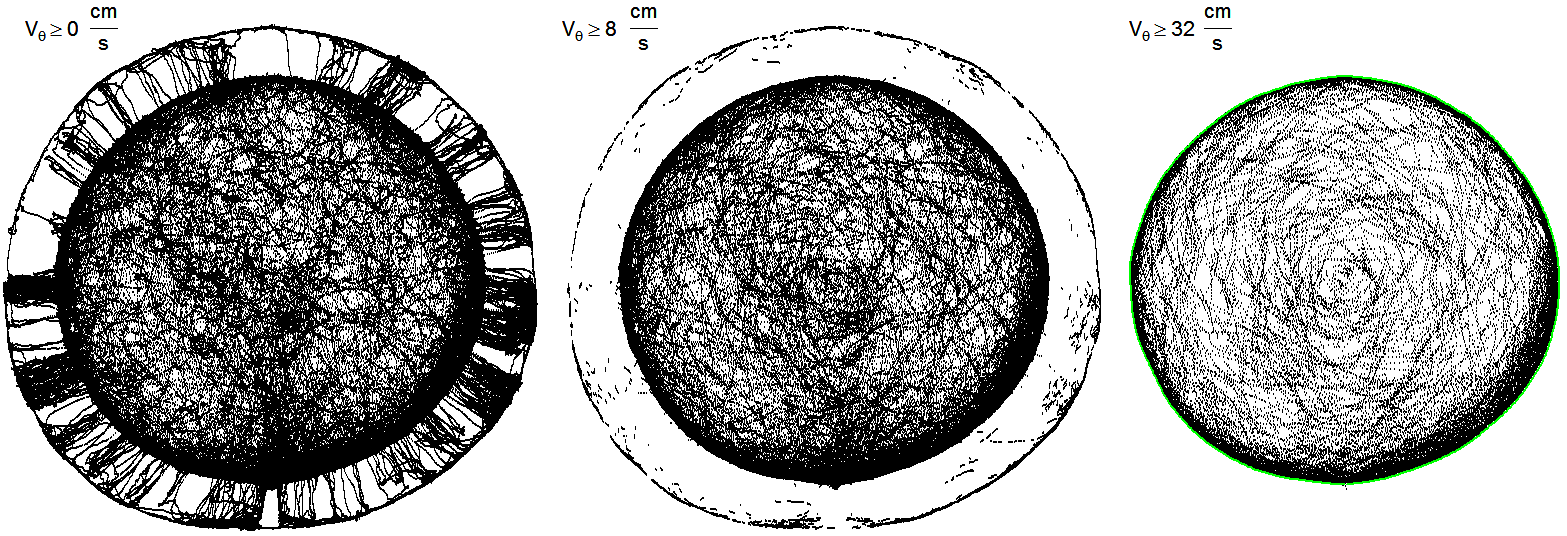

Supplement: Figure S3. High tangential speed indicates movement on the floor [file rsos180069supp3.tif]

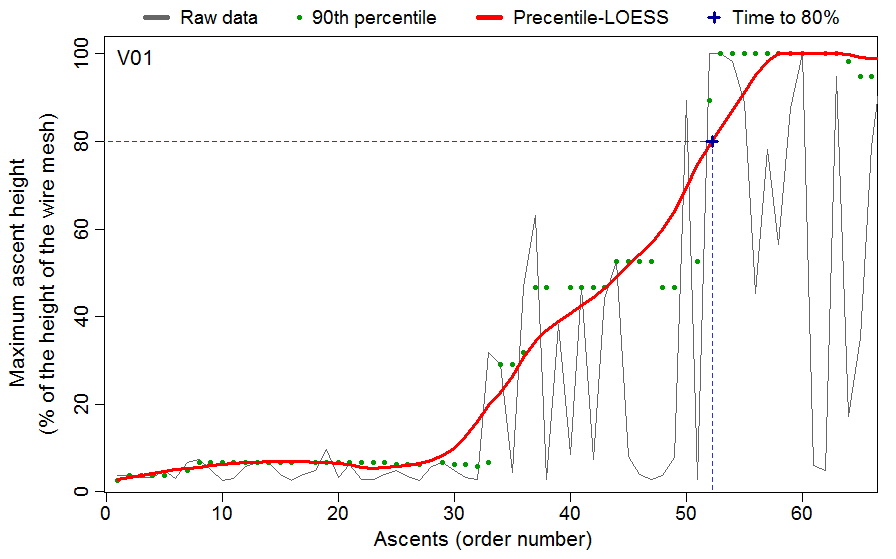

Supplement: Figure S4. Percentile-LOESS function estimates the growth in ascents' height [file rsos180069supp4.tif]

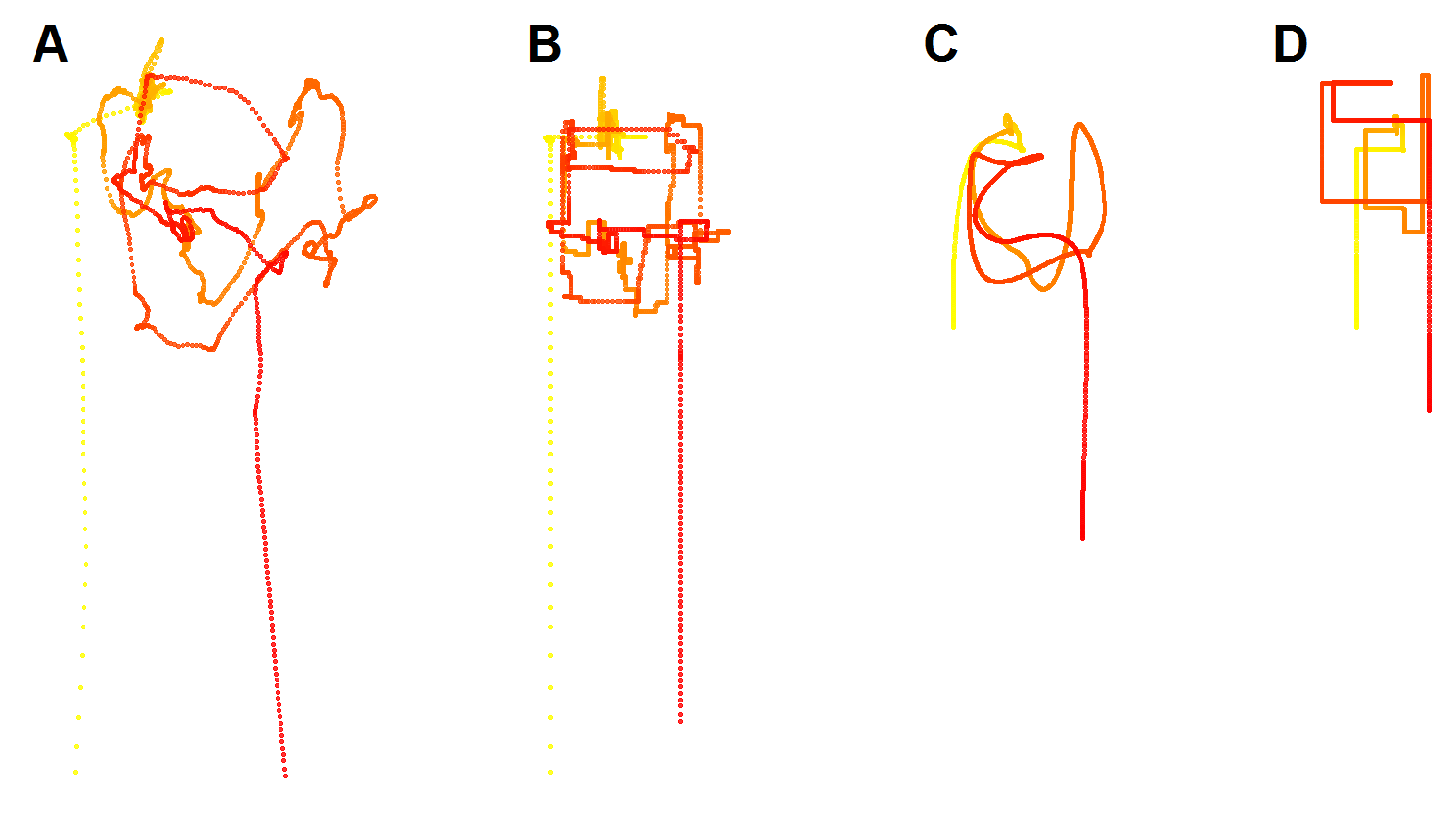

Supplement: Figure S5. Estimating the number of direction changes in an ascent [file rsos180069supp5.tif]
