# Supplementary material for: Vertical exploration and dimensional modularity in mice
Source: R Soc Open Sci. 2018 Mar 14;5(3):180069. doi: 10.1098/rsos.180069 (PMC5882751; doi:10.1098/rsos.180069)
Supplement: Supplementary videos [file rsos180069supp6.docx]

# Supplementary videos

# **Video S1**: **The morphogenesis of mouse exploratory behavior along the vertical dimension.**

<https://www.youtube.com/watch?v=5ecNKm_Jo0w&feature=youtu.be>

# **Video S2**: **The morphogenesis of mouse vertical exploration, centered on the point of ascent.**

# <https://www.youtube.com/watch?v=_wjW_1jlnp8&feature=youtu.be>

# **Video S3**: **Coverage of the lower circumference (point reference)**

# <https://www.youtube.com/watch?v=k8JSRM_I5Ow&feature=youtu.be>

# **Video S4**: **Coverage of the upper circumference (linear reference)**

# <https://www.youtube.com/watch?v=zQ-oSOrWLwQ&feature=youtu.be>

# **Video S5**: **Bouts of ascents and incursions in a BALB/c mouse (early entries)**

# <https://www.youtube.com/watch?v=HGepYFC1Wa0&feature=youtu.be>

# **Video S6**: **Bouts of ascents and incursions in a BALB/c mouse (late entries)**

<https://www.youtube.com/watch?v=qjRtxQT90HM&feature=youtu.be>
